# Central vein sign: comparison of multiple sclerosis and leukoaraiosis

**DOI:** 10.55730/1300-0144.5541

**Published:** 2022-09-21

**Authors:** Hüseyin Gökhan YAVAŞ, Ergin SAĞTAŞ

**Affiliations:** 1 Department of Radiology, Ahi Evran University Kırşehir Education and Research Hospital, Kırşehir, Turkey; 2 Department of Radiology, Faculty of Medicine, Pamukkale University, Denizli, Turkey

**Keywords:** Multiple sclerosis, leukoaraiosis, susceptibility-weighted imaging, central vein sign

## Abstract

**Background/aim:**

Leukoaraiosis produces white matter lesions (WML) similar to multiple sclerosis (MS) on brain magnetic resonance imaging (MRI), and the distinction between these two conditions is difficult radiologically. This study aimed to investigate the role of the central vein sign (CVS) in susceptibility-weighted imaging (SWI) sequence in distinguishing MS lesions from leukoaraiosis lesions in Turkish population.

**Materials and methods:**

In this prospective study, axial SWI and sagittal three-dimensional fluid-attenuated inversion recovery (3D-FLAIR) were obtained in 374 consecutive patients. The study consisted of 169 (89 MS patients, 80 patients with leukoaraiosis) patients according to the inclusion and exclusion criteria. Two observers evaluated MR images by consensus, and observers were unaware of the patient’s clinical findings. Locations (periventricular, juxtacortical, and deep white matter) and the presence of CVS were investigated for each of the lesions. Differences between patients in the leukoaraiosis and MS groups were investigated using the Mann-Whitney U test or chi-square analysis. In addition, receiver operating characteristic (ROC) analysis was used to analyze the diagnostic performance of CVS.

**Results:**

A total of 1908 WMLs (1265 MS lesions, 643 leukoaraiosis) were detected in 169 patients. The CVS was significantly higher in the MS lesions (p < 0.001). The CVS positivity rate in periventricular WMLs was higher than in juxtacortical WMLs or deep WMLs, both for all patients and for patients with MS (p < 0.001). The area under the curve (AUC) of the ROC analysis was 0.88 (95% confidence interval 0.83–0.93) for CVS in the distinction of MS lesions and leukoaraiosis.

**Conclusion:**

The presence of CVS in the SWI sequence can be used as an auxiliary finding for the diagnosis of MS in the differentiation of MS and leukoaraiosis lesions.

## 1. Introduction

Multiple sclerosis (MS) is a chronic, neurodegenerative disease characterized by multiple inflammatory demyelinating lesions of the central nervous system (CNS). This condition is the most common cause of nontraumatic disability in young adults [1,2] and is pathologically characterized by multifocal inflammation, demyelination, axonal damage, and neuronal loss [3]. There is no specific diagnostic test for MS [4]. Instead, this diagnosis is achieved by evaluating a patient’s clinical characteristics, laboratory results, electrophysiological studies, and imaging results. For the latter, magnetic resonance imaging (MRI) is the most used and most effective method in radiological evaluation. The McDonald criteria based on MRI have facilitated early diagnosis and initiation of disease-modifying treatment, significantly improving disease outcomes. However, these criteria have not achieved sufficient sensitivity or specificity [5,6]. Vascular, infectious, neoplastic, congenital, and metabolic diseases may cause misdiagnosis by causing lesions that can mimic MS on MRI [7–9]. Therefore, the distinction between MS lesions and leukoaraiosis is critical.

Various studies have been conducted to distinguish MS lesions from other pathologies that produce similar signal changes on MRI. An MRI detectable central vein sign (CVS) in white matter lesions (WML) has been proposed as a biomarker of inflammatory demyelination, and CVS has been suggested as an auxiliary finding in MS lesion diagnosis [10]. Histopathological postmortem studies have indicated that the localization and shape of MS lesions are appropriate for perivenular distribution, supporting CVS in MRI [2,4,11]. Researchers have attempted various MRI sequences to reveal the perivenular localization of MS lesions. One such sequence is susceptibility-weighted imaging (SWI), a flow-compensated gradient-echo sequence that can detect the vein in the center of the lesion due to its high sensitivity to slow venous flow [1,12,13]. Recent studies comparing the presence of CVS in different neurological diseases and MS lesions have shown that CVS can accurately distinguish MS from mimickers [14–16]. However, more research in different populations is needed for CVS to be accepted as a reliable diagnostic criterion. One of the common differential diagnoses for MS is focal white matter hyperintensity (leukoaraiosis) due to cerebral small vessel disease (CSVD). Therefore, this study compares the presence of CVS in WMLs in MS patients and the presence of CVS in patients with leukoaraiosis and the distribution of CVS-positive lesions across locations in the Turkish population.

## 2. Materials and methods

This study has a prospective character, and the researchers began this study after receiving approval numbered 60116787-020/8901 dated 06.02.2019 from the “Pamukkale University Non-Interventional Clinical Research Ethics Committee”. After the team informed the patients about the test and study, they obtained the participants’ written and verbal consent. Additionally, MRI safety rules were observed before the patients were taken into the device, and the patients were not allowed to participate in the presence of any contraindications.

### 2.1. Case selection

This study included 374 patients scheduled for brain MRI scans between February 15, 2019, and August 15, 2019. These patients exhibited no malignancy, vasculitis, rheumatologic disease, or neurological disease other than MS. Beyond this, no patient has a history of previous cerebrovascular accident (CVA) or cranial operation.

Of these participants, 177 were followed up with a diagnosis of MS in the hospital’s neurology clinic or their initial MS diagnosis using clinical and radiological findings. All these patients met the dissemination in space criteria according to the 2017 McDonald criteria. Of these 177 patients, 19 had hypertension and 15 had diabetes. The MR image quality of 16 patients was insufficient for evaluation, and 38 patients did not have lesions suitable for evaluation (Table 1). Eighty-eight patients were excluded from the study for these reasons, and the remaining 89 patients (35 men, 54 women, mean age 38, range 18–64) were called the MS group.

**Figure 1:**
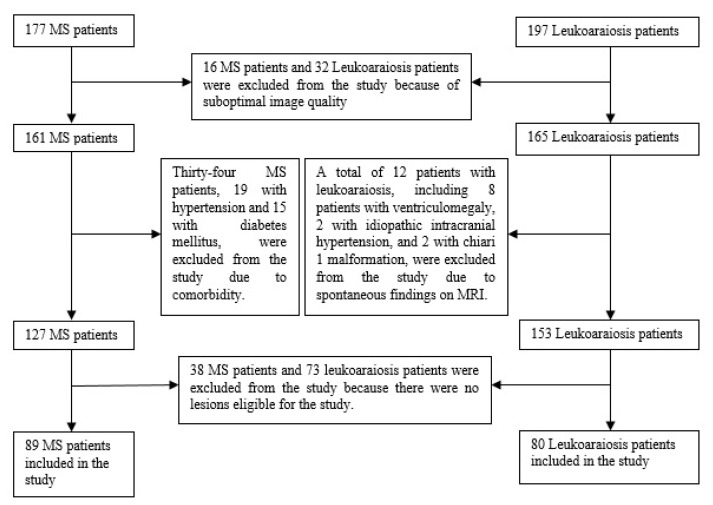
Diagram of patients excluded and included in the study.

One hundred and ninety-seven patients had no MS and other neurological diseases, oncological diseases, past CVA and had no complaints other than nonspecific ones such as headache, dizziness, or weakness. The quality of the images from 32 of these patients was suboptimal, and the appropriate lesion (Table 1) was not observed in 73 of them. Beyond this, previously unknown Chiari 1 malformation, idiopathic intracranial hypertension, or ventriculomegaly were detected on MRI images from 12 patients. For these reasons, 117 patients were excluded from the study. The remaining 80 patients (31 men and 49 women, mean age 56, range 33–65) constituted the second group, the Leukoaraiosis group. The diagram of patients included and excluded from the study appears in Figure 1.

### 2.2. MRI protocol

Cranial MRI examinations of the patients included in the study were performed using a neurovascular dStream digital head coil in a 1.5 Tesla superconducting magnet (Philips Ingenia; Philips Medical Systems, Best, Netherlands) system at the hospital. Furthermore, the MRI examination protocol, sagittal 3D FLAIR (Repetation time: 4800 ms, echo time: 366 ms, matrix: 252 mm × 184 mm, slice thickness: 0.9 mm, slice gap: 0 mm, FOV: 230 mm × 167 mm, inversion time: 1660 ms), and axial SWI (Repetation time: 35 ms, echo time: 30 ms, matrix: 256 mm × 197 mm, slice thickness: 2 mm, slice gap: 0 mm, FOV: 230 mm × 167 mm) sequences were added to routine imaging in all patients. MRI protocol and sequence properties are given in Table 2.

### 2.3. Image evaluation

In this study, MRI data were analyzed by a 20-year experienced neuroradiologist and a radiologist with four years of experience. Both of these researchers were blinded to the patients’ clinical data. Axial 2D-FLAIR and sagittal 3D-FLAIR sequences were used to identify lesions of MS or leukoaraiosis in the supratentorial white matter in each patient. Infratentorial lesions, cortical lesions, and brainstem lesions can be only partially evaluated because of their proximity to bone structures and mastoid cells, leading to significant susceptibility artifacts in SWI sequences [1,2,17]. Therefore, these lesions were not included in this study.

**Figure 2:**
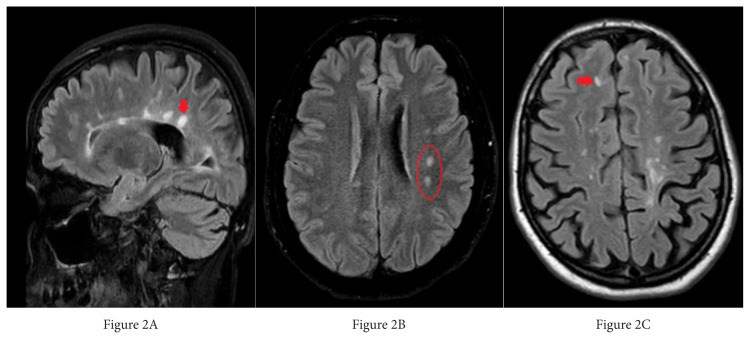
White matter lesion locations: A) Sagittal 3D-FLAIR sequence, periventricular lesion, B) Axial 2D-FLAIR sequence, deep white matter lesion, C) Axial 2D-FLAIR sequence, juxtacortical lesion.

**Figure 3:**
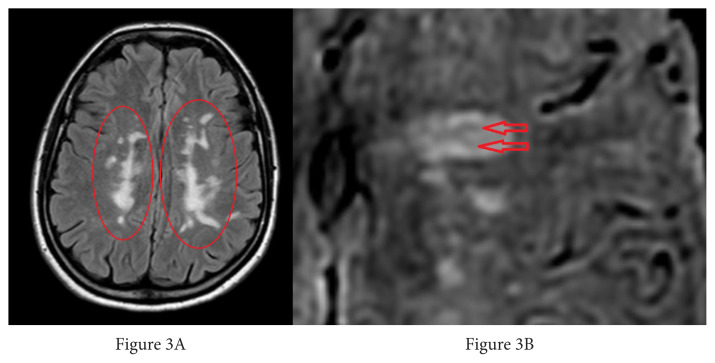
Examples of excluded lesions: A) White matter lesions with a bilateral tendency to merge were observed in the axial FLAIR image, and these lesions were excluded from the study. B) Axial SWI image, the lesion was not included in the study due to more than one hypointensity in the center of the periventricular white matter lesion located in the left frontal lobe.

Each lesion was first identified in 3D FLAIR images. To evaluate the CVS according to the location of each lesion, the researchers examined these lesions by dividing them into three locations: periventricular (those touching the ventricle wall), juxtacortical (those touching the cortex), and deep white matter (all remaining supratentorial WMLs) [18,19] (Figures 2a, 2b, and 2c). The inclusion criteria for WMLs in this study appear in Table 1 [16] (Figures 3a and 3b).

**Figure 4:**
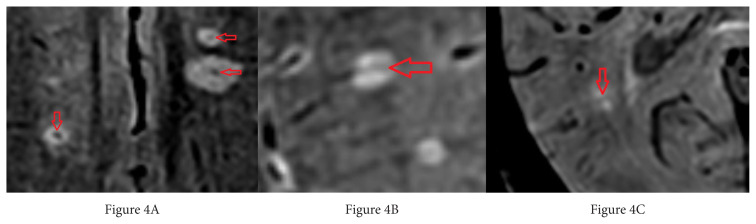
A) Axial SWI view, point hypointensity consistent with CVS in the center of deep white matter lesions with two posterior left frontal lobes and one posterior to the right frontal lobe. B) Axial SWI image, linear hypointensity parallel to the long axis of the lesion in the center of the white matter lesion located posterior to the left frontal lobe was evaluated in favor of the CVS. C) Axial SWI image, although the deep white matter localized lesion in the right temporal lobe has hypointensity in the center, this lesion was considered CVS negative because it was located perpendicular to the long axis of the lesion.

After the WMLs were identified, the presence of CVS in the SWI sequence was investigated. In the SWI sequence, the presence of a hypointense point in the center of the round lesion or a linear hypointensity parallel to the long axis of the ovoid lesion, and crossing the center of the lesion (Figures 4a and 4b) was accepted as CVS [16]. Any hypointensities lacking these features were not accepted as CVS (Figure 4c). It was based on the consensus of both observers in the evaluation of whether the lesions were included in the study and the presence of CVS in the lesions. After determining whether each lesion was positive or negative for central vein sign, the central vein sign positivity rate was determined by dividing the number of central vein sign positive lesions by the total lesion number for each patient. Then, statistical analysis was performed on these ratios.

### 2.4. Statistical analysis

The data were analyzed using the SPSS 25.0 package program. Continuous variables are given as mean, minimum, and maximum values, and categorical variables are displayed as numbers and percentages. Next, the Mann-Whitney U test was used to compare independent group differences, and a chi-square analysis was used to compare categorical variables. Additionally, the ROC analysis method was employed to examine the performance and validity of the CVS positivity rate measurements. From the ROC analysis, the Youden Index value was used to determine the most appropriate cut point. Sensitivity, specificity, positive predicted value, and negative predicted value were obtained from the examinations involving the most appropriate cut-off points obtained according to CVS positivity rate results. Finally, the performance results were examined.

## 3. Results

### 3.1. Demographic features

In this study, the researchers evaluated MR images of 89 patients (mean age 38.4, range 18–64 years) in the MS group and 80 patients (mean age 55.6 years, range 33–65 years) in the leukoaraiosis group. Overall, there were more female than male patients in both groups (60.7% in the MS group and 61.2% in the leukoaraiosis group). The age and gender distributions of the patients are shown in 3.

### 3.2. Number and distribution of WMLs

In this work, 1908 WMLs were evaluated, including 1265 WMLs in the MS group and 643 WMLs in the leukoaraiosis group. All lesions were selected in axial 2D-FLAIR, sagittal 3D-FLAIR, and SWI sequences.

When the locations of the lesions were examined, most of the lesions in the MS group were observed in the periventricular area (612 lesions, 48%). In the leukoaraiosis group, most of the lesions were in deep white matter (449 lesions, 70%). The distribution of the lesions examined according to groups and locations is summarized in Table 3.

In the patients in the MS group, the number of periventricular lesions was higher than the number of deep white matter lesions (p < 0.001), and the number of juxtacortical lesions (p < 0.001). Beyond this, there was no statistically significant difference between the number of deep white matter lesions and the number of juxtacortical lesions (p = 0.072).

In the leukoaraiosis group, the number of deep white matter lesions was statistically significantly higher than the number of periventricular lesions (p < 0.001) and the number of juxtacortical lesions (p < 0.001). In this group, no statistically significant difference was found between the periventricular white matter and the number of juxtacortical lesions (p = 0.443).

### 3.3. Evaluation of CVS

**Figure 5:**
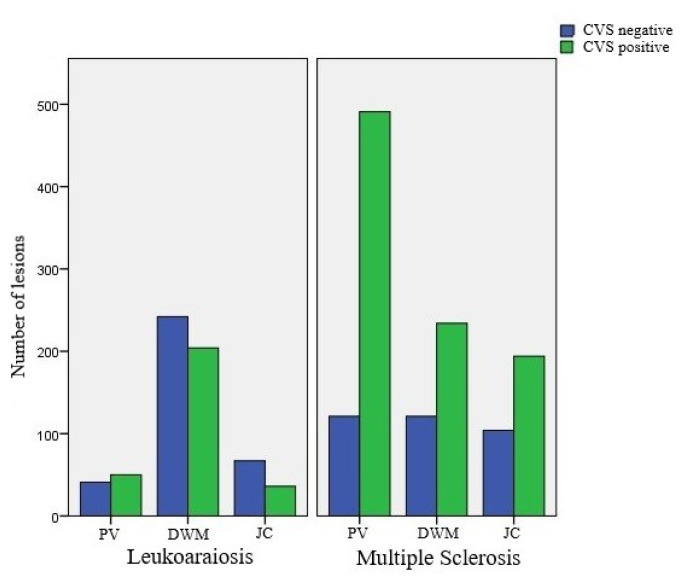
Schematic representation of the distribution of white matter lesions and CVS positivity rates by location in both patient groups (PV: Periventricular, DWM: Deep white matter, JC: Juxtacortical, CVS: Central vein sign).

Subsequently, CVS was positive in 919 (72.6%) of the MS group lesions and 290 (45.1%) lesions from the leukoaraiosis group. There was no significant relationship between CVS rates and age or gender in either group. Additionally, the distribution of CVS positivity rates of the lesions is shown schematically in Figure 5.

The CVS positivity rates in periventricular WMLs and in the MS group were higher than those of deep white matter lesions (p < 0.001) and juxtacortical lesions (p < 0.001). There was no significant difference between CVS positivity rates of lesions in deep white matter and juxtacortical locations (p = 0.114 in total, p = 0.807 in the MS group). In the leukoaraiosis group, no statistically significant difference was found between the CVS positivity rates of the lesions in any of the three locations. The distribution of CVS-positive lesions in the MS and leukoaraiosis groups by location is shown in Table 3.

The CVS positivity rate in all WMLs in patients in the MS group was higher than in patients with leukoaraiosis (p < 0.001). Additionally, in the comparisons between both groups in the periventricular white matter, deep white matter, and juxtacortical areas; higher CVS was found in MS patients than in patients with leukoaraiosis (Table 3). When all locations were evaluated together, the area under the curve was calculated as 0.88 (95% confidence interval 0.83–0.93) in the ROC (receiver operating characteristic) analysis. According to the results of the ROC analysis, when the cut-off point of CVS positivity in WMLs was determined to be 59%, the sensitivity for CVS in the diagnosis of MS was 84.2%, the specificity was 77.5%, the positive predictive value was 80.6%, and the negative predictive value was 81.5%.

## 4. Discussion

Multiple sclerosis is a central nervous system disease that causes neurological deficits, especially in young adults, so it is essential to distinguish MS from other central nervous system diseases. However, for this, additional findings are needed to strengthen the current MS diagnostic criteria. Histopathological postmortem studies indicated that the localization and shape of MS lesions were appropriate for perivenular distribution. MRI-detectable central vein sign (CVS) in white matter lesions (WML) has been proposed as a biomarker of inflammatory demyelination and may therefore aid the diagnosis of MS. The literature already includes studies examining CVS in MS. Most of these studies have been conducted with a 3T MRI device, and in recent years, few studies have been conducted with a 7T MRI device. However, the number of studies performed with a 1.5T MRI device has been relatively small. This study compared the CVS rates from MS patients and patients with leukoaraiosis using a 1.5T MRI device. Additionally, 1908 WMLs were evaluated in 169 patients, 89 of whom were MS patients and 80 of whom were leukoaraiosis patients. The numbers of patients and lesions were higher than those in many studies in the literature. Furthermore, in this study, the CVS rate in the MS group was 72.6%, while this rate was 45.1% in the leukoaraiosis group. According to these results, the CVS positivity rate in MS patients was higher than in patients with leukoaraiosis in the Turkish population, a finding consistent with many studies in the literature [1,14,20–24].

Similarly, one study in the literature compared CVS in MS and control groups using a 1.5T magnetic power device, and the results were published by Sparacia et al. in 2018 [24]. In this work, FLAIR and SWI sequences were used to evaluate the WML and CVS. There were 19 patients in each group, and the study evaluated 313 lesions in the MS group and 75 lesions in the small vascular access group. According to the results of this study, CVS positivity was 40.9% in the MS group. In the present study, this rate was higher: 72.6%. In the group with small vascular disease, the CVS positivity rate was 29.3%, but in the current study, this rate was 45.1%. In the study by Sparacia et al., the rate of CVS positivity in lesions of MS patients was higher than that of patients with cerebral small vascular disease, results consistent with the present study. Beyond this, the number of patients included in this study was higher than the number of patients included in the study by Sparacia et al. Additionally, unlike in the work of Sparacia et al., this study did reveal a 3D-FLAIR sequence, and other studies have indicated that this method is superior to 2D-FLAIR sequences in detecting lesions in MS patients [25]. The higher number of CVS in both groups in the present study than in the other study mentioned may have resulted because the current team detected more lesions in more patients by using 3D-FLAIR than the researchers in the other study. Additionally, MS patients with diseases such as diabetes and hypertension that could cause WMLs of leukoaraiosis were excluded from the current study. On the other hand, in the study by Sparacia et al., no such exclusion criterion was mentioned and therefore some lesions observed in MS patients may result from CSVD, producing a low CVS rate.

In a study by Maggi et al. in 2018, MS and inflammatory vasculopathies were compared in terms of CVS positivity [26]. This multicenter study used 1.5T and 3T devices and employed FLAIR and T2* EPI (echo-planar imaging) sequences following intravenous contrast material administration for CVS evaluation. The study evaluated 52 MS patients and 31 inflammatory vasculopathy patients [26]. Overall, the CVS rate in the MS group was 88%, whereas the CVS rate in the inflammatory vasculopathy group was 14% and higher in MS patients than in the other group. This finding was consistent with the current study.

A metaanalysis published in 2019 by Suh et al. [27] reviewed CVS, evaluating 21 studies reporting outcomes for 501 MS patients. In the studies evaluated in this meta-analysis, the rate of CVS in MS patients varied between 40% and 90%, and the average rate of CVS in MS lesions was 74% [27]. Again, in the metaanalysis published by Castellaro et al. in 2020, this rate was 73% [28]. In the present study, the rate of CVS in MS patients was 72.6%, consistent with the results of the other studies mentioned.

Additionally, the metaanalyses published by Suh et al. [27] and Castellaro et al. [28] examined the CVS differences according to the magnetic strength of the MRI devices used in the studies. The CVS rate increased in direct proportion to the magnetic power of the device, especially in the MS group. In the studies performed with 7T, 3T, and 1.5T devices, the mean CVS rates were 84%, 79%, and 56%, respectively, in the study by Suh et al., and 82%, 74%, and 58%, respectively, in the study by Castellaro et al. In the metaanalysis by Suh et al., 11 studies had a control group and used 3T and 7T devices. In these studies, the CVS rate of the control group decreased as the magnetic strength increased. However, this rate was not statistically significant (26% in studies with 7T devices and 38% in studies with 3T devices). Compared with the studies performed with 3T and 7T MRI devices, this study may have produced a lower CVS rate in the MS group and a higher CVS rate in the control group (leukoaraiosis) because this work used a 1.5T MRI device. However, these results could change depending on the number of patients, their demographics, and control group diseases. Beyond this, the cut-off values in CVS rates in the studies included in the metaanalysis by Suh et al. varied between 30% and 67%, but these rates varied between 30% and 54% in the studies included in the metaanalysis by Bhandari et al. in 2020 [29]. After the statistical evaluation in the metaanalyses, the most appropriate cut-off values were 40% [27] and 45% [28]. In this study, the cut-off value was 59%, a high value because the CVS rate in the leukoaraiosis group was higher than the averages stated in the metaanalysis.

Consistent with many studies in the literature, this study revealed a higher CVS rate in the WMLs of MS patients compared to the WMLs of control patients (leukoaraiosis). However, there were differences in the CVS percentages of lesions between the MS and control groups, both among the publications in the literature and in the present study. While CVS positivity rate in MS lesions is over 80% in some publications [1,5,20,26,30], it has been reported as 40%–50% in some publications [21,24,31]. Close values were reported in the present study and in the study of Mistry et al. (72%, 72.7%, respectively). There may be many reasons for this difference. First, in many of these studies, the magnet power of the MRI device was 3T, whereas fewer studies had a magnet power of 1.5T. As this power decreases, the image quality decreases and the evaluation of the lesions becomes difficult, so the CVS detection rate may decrease accordingly [27]. Second reason for the aforementioned discrepancies between studies may be the differences in the detection rates of WMLs depending on whether 3D-FLAIR was used or not. Third, in the literature, five different sequences, SWI, SWAN, T2*, T2* EPI, and FLAIR*, were used to detect CVS, and this detection efficiency may vary, causing the differences noted among the results discussed. Fourth, the present study did not include lesion sizes, and the literature has demonstrated that as the size of the WML increases, the rate of CVS detection increases [1]. Therefore, if the lesions in the current study were smaller than the lesions included in the studies in the literature, a lower CVS rate may have resulted. In addition to these, studies have been conducted regarding the effect of the ages of MS lesions on the detection of CVS [32,33]. According to a view, there may be a decrease in deoxygenized hemoglobin in the venous structure in the center due to hypometabolic conditions in chronic stage plaques, thus decreasing the detection of SWI sequences. The ages of the lesions were not investigated in this study, so one of the reasons for the difference in CVS rates may be lesion ages.

Additionally, the WMLs in MS are more likely to be visible in the periventricular area than in other diseases [34]. Especially in the ventricular neighborhoods, the venous system displays more intensity, so CVS could be seen more in these regions [17]. In the present study, lesions were examined from three groups: periventricular, deep white matter, and juxtacortical. In the MS group, the CVS rate (80.2%) of lesions in the periventricular space was higher than the rate in the other two locations (65.9% in the deep white matter and 65.1% in the juxtacortical area). There was no statistically significant difference between CVS rates in all three locations in the other group. The reason for this difference may be that there are more venous structures in the periventricular space and MS lesions are more common in the periventricular space. Since leukoaraiosis lesions are mostly arteriole-based [35], the distribution of venous structures within the parenchyma should not affect the distribution of the CVS rate in WMLs due to leukoaraiosis.

According to the results of this study, the rate of CVS in MS patients in the Turkish population was found to be higher than in patients with leukoaraiosis, similar to studies conducted in different populations in the literature. Additionally, the CVS rate in lesions in the periventricular area was higher in MS patients than that in other locations.

However, the current study had several limitations. For instance, this study was conducted in a single center. Apart from this, the decrease in the number of patients as a result of low quality MR images, which are generally caused by patient-related problems, can be counted among the limitations.

## Figures and Tables

**Table 1:** Inclusion criteria for white matter lesions in the study.

The long diameter of the lesion ≥ 3 mm
The lesion does not tend to merge with another white matter lesion.
Not having more than one hypointensity in the lesion.
Lesion borders can be seen in both FLAIR and SWI sequences.

FLAIR: Fluid attenuated inversion recovery, SWI: Susceptibility weighted imaging

**Table 2:** Sequences and details in the MRI protocol.

	TR (msn)	TE (msn)	Matrix	Slice thickness (mm)	Slice gap (mm)	FOV (mm × mm)	TI (msn)	Epi factor	b value (s/mm^2^)
**Ax T1 MTC**	535	15	272 × 160	5	1	183 × 131			
**Ax T2**	6165	110	205 × 256	3	1	230 × 179			
**FLAIR**	11,000	140	256 × 168	3	1	193 × 159	2800		
**DWI**	3231	85	105 × 192	5	1	230 × 179		53	0 and 1000
**SWI**	35	30	256 × 197	2	0	230 × 179			
**Sag 3D FLAIR**	4800	366	252 × 184	0.9	0	230 × 167	1660		

MS: Multiple sclerosis, Ax: Axial, Sag: Sagittal

**Table 3:** Demographic characteristics of patients in MS and leukoaraiosis groups, numbers and percentages of white matter lesions in patients and distribution of CVS-positive lesions by location.

	MS	Leukoaraiosis	P-value
Number of patients (percentage)	89	80	0.939
Male	35 (39.3)	31 (38.8)	
Female	54 (60.7)	49 (61.2)	
Mean age	38.4	55.5	<0.01
Number of lesions (percentage)	1265	643	<0.01
Periventricular	612 (48)	91 (14)	<0.01
Deep white matter	355 (28)	449 (70)	0.08
Juxtacortical	298 (24)	103 (16)	<0.01
Number of CVS positive lesions (CVS positivity percentage)	919 (72.6)	290 (45.1)	<0.01
Periventricular	491 (80.2)	50 (54.9)	<0.01
Deep white matter	234 (65.9)	204 (45.4)	0.08
Juxtacortical	204 (65.1)	36 (34.9)	<0.01

CVS: Central vein sign, MS: Multiple sclerosis
